# A Giant Fibroepithelial Polyp of the Nipple: A Case Report of a Rare Benign Tumor

**DOI:** 10.7759/cureus.108537

**Published:** 2026-05-09

**Authors:** Saif S Al Sahee, Jaideep Rait, Layloma Hamidi-Latifi

**Affiliations:** 1 Department of Breast Surgery, Maidstone and Tunbridge Wells NHS Trust, Maidstone, GBR

**Keywords:** acrochordons, benign breast tumor, fibroepithelial polyp, giant, histopathology, nipple lesion

## Abstract

Fibroepithelial polyps (FEPs) are rare benign tumors that most commonly arise in the lower female genital tract, including the vagina, vulva, and cervix. Occurring in the breast, particularly the nipple, is extremely rare and may clinically and radiologically mimic malignant disease. A woman in her early 50s presented to the breast one-stop clinic with a progressively enlarging pedunculated lesion arising from the nipple. She reported a longstanding small nipple tag since childhood that had recently increased significantly in size. Clinical examination revealed a 9 x 7 cm soft, exophytic, pedunculated mass arising from the nipple. Breast imaging showed no underlying abnormality. The patient underwent day-case surgical excision under general anesthesia with preservation of the nipple and uncomplicated recovery. The specimen weighed 130 g. Histopathology demonstrated a fibrovascular lesion with dense collagenous stroma and overlying hyperplastic epithelium, without atypia or malignancy. FEP of the nipple is an exceptionally rare entity that should be considered in the differential diagnosis of nipple lesions. Surgical excision is curative and allows excellent cosmetic and functional outcomes.

## Introduction

Fibroepithelial polyps (FEPs), also known as acrochordons, are benign mesodermal tumors that most commonly arise in the lower female genital tract, particularly in the vagina, and less frequently in the vulva and cervix [1,2]. Their occurrence in the breast, especially in the nipple, is exceedingly rare, with only 10 isolated cases reported in the literature [3].

FEPs are either solitary or multiple, usually a few millimeters in size. The precise etiopathogenesis of the FEPs remains uncertain; however, they are believed to develop either secondary to focal loss of elastic tissue or slow-growing hamartoma or fibrous lesions comprising a variety of tissue components [4].

Although FEPs are benign lesions, they might pose a diagnostic challenge due to their morphological overlap with a range of mesenchymal tumors, including malignant entities such as sarcomas. They can share histological features with lesions such as angiofibroblastoma, aggressive angiomyxoma, cellular angiofibroma, leiomyoma, perineuroma, and neurofibroma [2]. We report a rare case of a giant FEP arising from the nipple in a woman in her early 50s.

## Case presentation

A female patient in her early 50s presented to the breast one-stop clinic with a painless, progressively enlarging, pedunculated, exophytic lesion arising from the left nipple. She reported a longstanding nipple tag present since childhood, which had recently increased significantly in size, prompting her to seek medical attention. Her past medical history included asthma, managed with an inhaler. She was nulliparous, perimenopausal, and not on hormonal replacement therapy. Her BMI was 26.7, and there was no family history of breast cancer.

**Figure 1:**
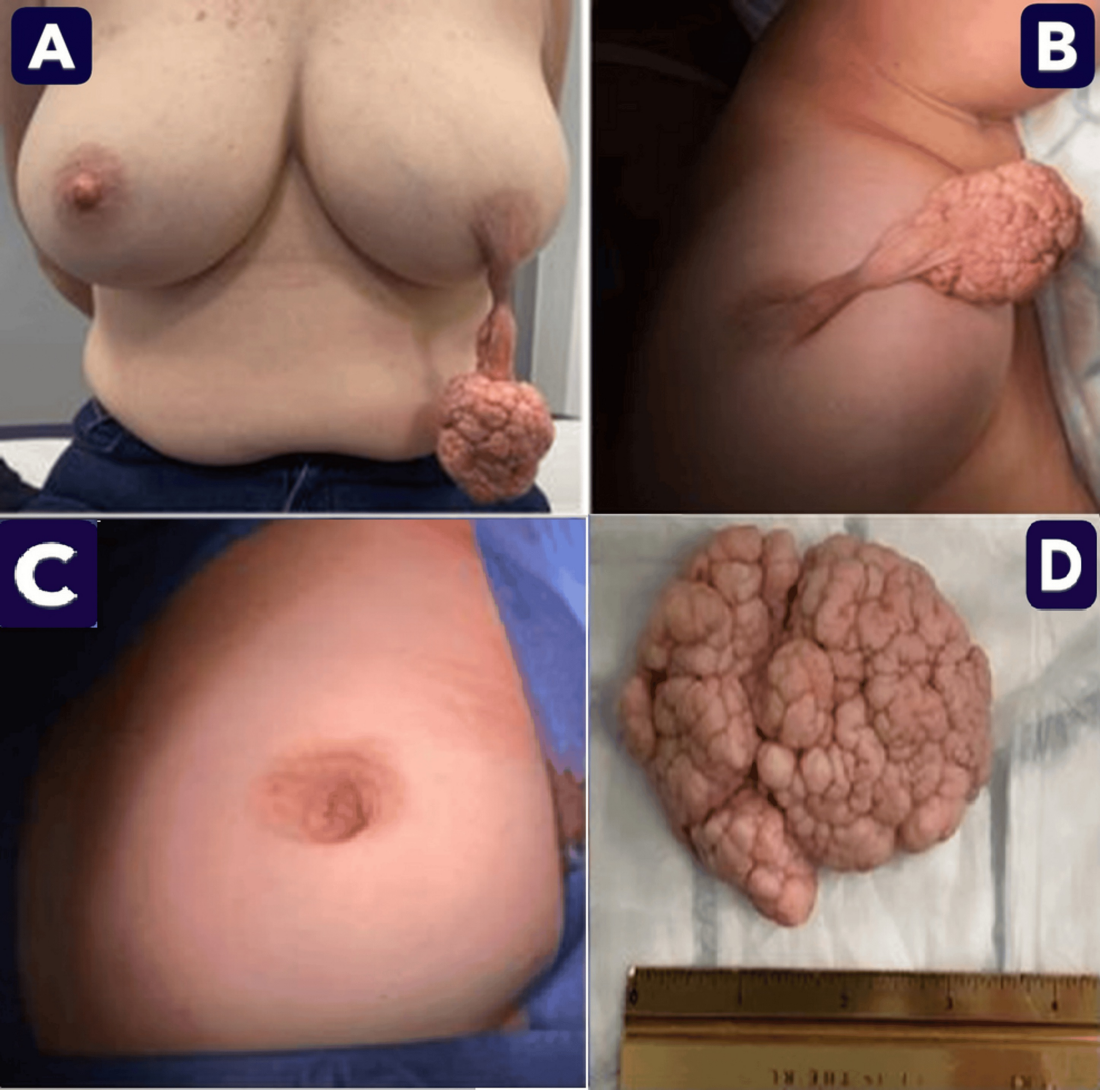
Clinical and gross features of the fibroepithelial polyp (A) Frontal clinical view demonstrating a large pedunculated mass arising from the left nipple-areolar complex. (B) Elongated stalk and lobulated contour of the lesion. (C) Postexcision appearance of the nipple-areolar complex. (D) Gross specimen showing the mass postexcision

On clinical examination, her only abnormality was a 9 × 7 cm pedunculated lesion extending from the left nipple. The lesion was firm and lobulated, with normal overlying skin and no hyperpigmentation, and was connected to the nipple by a soft, pliable stalk. No other abnormalities were detected (Figure 1).

**Figure 2:**
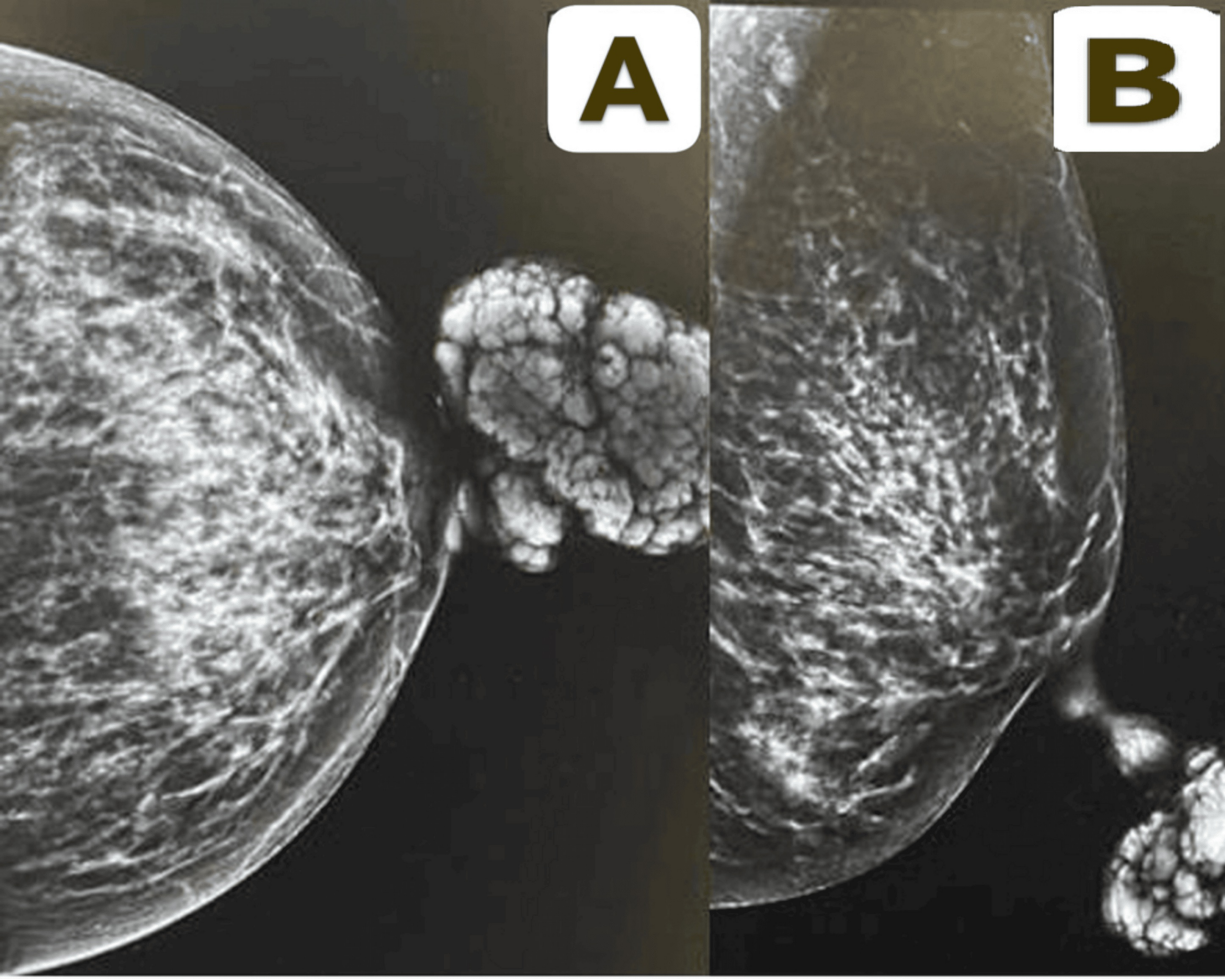
Left breast mammogram showing the 9 x 7 cm pedunculated lesion attached to the nipple. (A) Left breast craniocaudal view. (B) Left breast mediolateral oblique view

Baseline breast imaging demonstrated no suspicious underlying pathology aside from the lesion itself, which measured 9 × 7 cm (Figure 2). This represented an increase in size compared with prior imaging from 2017, when it measured 6 × 6 cm. The mammographic images demonstrate a well-defined, pedunculated, exophytic mass arising from the left nipple. The lesion appears lobulated, with a cauliflower-like surface contour projecting externally from the nipple-areolar complex. It shows homogeneous soft-tissue density without internal calcifications or spiculated margins. Importantly, there is no associated architectural distortion, skin thickening, or underlying parenchymal abnormality within the breast tissue. The surrounding breast parenchyma appears unremarkable, supporting a benign etiology.

The patient was counseled on surgical excision, including the indications and potential complications of the procedure. She subsequently underwent an elective day-case surgical excision under general anesthesia. The procedure was uncomplicated, performed with an elliptical incision and wound closure with 3/0 Monocryl sutures. She was discharged on the same day, and no further treatment was required.

**Figure 3:**
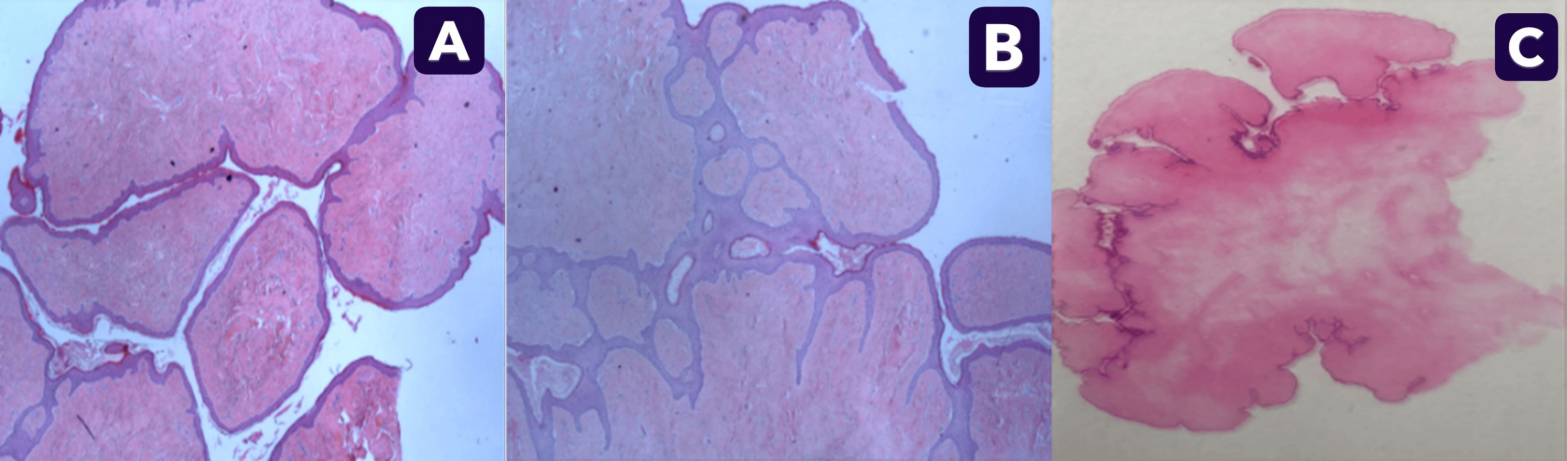
Histopathological features of fibroepithelial lesion (A) Low-magnification view (×40) demonstrating overall lobulated architecture and fibrovascular core clearly. (B) Low-magnification view (×100) showing details of the stromal components and epithelial covering. (C) A broad section (×10) revealing the lobular architecture with surface hyperkeratosis

The excised specimen weighed 130 g. Histopathological evaluation, performed across six tissue blocks due to the lesion size, confirmed the diagnosis of a giant FEP of the nipple. Microscopic examination demonstrated a central core composed of dense collagen, with preservation of normal dermal structures, including blood vessels. No glandular components were identified (Figure 3).

Further histopathological assessment revealed a polypoid lesion with a lobulated architecture composed of fibrovascular cores covered by benign squamous epithelium. The stroma was edematous and contained scattered spindle-shaped stromal cells without cytological atypia. There was no significant mitotic activity, necrosis, or evidence of malignancy. The overlying epithelium was unremarkable, with no dysplasia. Surgical margins were clear. Immunohistochemical studies were not performed. Overall, the histological features were consistent with a benign fibroepithelial stromal polyp. At follow-up, she was reviewed in the clinic, informed of the histological findings, and expressed satisfaction with the outcomes.

## Discussion

Fibroepithelial lesions (FEPs), also known as acrochordons, were first described by Norris and Taylor in 1966 [5]. They typically occur in women of reproductive age and most commonly arise in the vagina, followed by the vulva and cervix. In contrast, FEPs involving the breast are exceedingly rare. FEPs are typically pedunculated lesions with a variable size spectrum. Smaller lesions measuring only a few millimeters are commonly referred to as skin tags, whereas lesions approaching approximately 5 cm are classified as FEPs. Those exceeding 5 cm in diameter, which are rare, are considered giant FEPs; the largest reported FEP was in the vulva, measuring 32 x 29 cm [6].

We reported a case of a fibroepithelial lesion arising from the nipple in a woman in her 50s, managed with surgical excision while preserving the nipple. This case is noticeable for both its unusual anatomical location and its exceptionally large size. The lesion was initially asymptomatic and small, and the patient did not seek medical attention until a recent rapid increase in size, eventually reaching 9 x 6 cm.

FEPs of the nipple described in the literature are typically small and detected early due to their visible location, sudden onset, or associated symptoms such as friction or bleeding [4]. A literature review was performed using the PubMed database up to February 2026 using the search terms "giant", "fibroepithelial polyp", and "nipple". Only English-language case reports and case series describing FEPs involving the nipple were included, and no studies meeting these criteria were excluded. The search identified nine relevant published cases. Among these, the largest documented lesions included a 4 x 4 cm lesion in a 45-year-old woman, a 3 x 2 cm lesion in a 65-year-old woman, and a 6 cm lesion in a 23-year-old patient [1,2,7]. To our knowledge, the present case represents the largest FEP lesion of the nipple reported in the literature to date. However, additional unpublished or nonindexed cases may exist.

Systematic conditions have been associated with FEPs, such as obesity, diabetes mellitus, and insulin resistance, and metabolic disturbances in carbohydrate and lipid pathways have been suggested as contributing factors. Human papillomavirus, particularly serotypes 6 and 11, has also been implicated in their development [8]. Further, a hormonal basis has been suggested, supported by the expression of estrogen and progesterone receptors within stromal cells. This theory is reinforced by their increased incidence during pregnancy and among postmenopausal women receiving hormone replacement therapy [9].

Histologically, FEPs are composed of a fibrovascular core covered by a hyperplastic epithelial surface. The underlying stroma typically shows a polypoid proliferation with variable morphological features, due to the presence of occasional atypical stromal cells; these lesions may be misdiagnosed as malignant entities, including sarcoma, or confused with benign but histologically similar tumors such as angiomyofibroblastoma, myofibroblastoma, and aggressive angiomyxoma, particularly when a myxoid stromal background is present [4,10]. This diagnostic challenge explains why FEPs were histologically referred to as “pseudosarcoma botryoides” [3]. In our case, histological examination demonstrated features consistent with an FEP, including prominent collagen deposition and absence of cellular atypia or malignant features.

Small FEPs are commonly managed with minimally invasive techniques such as cryotherapy or cauterization, whereas larger lesions are typically treated with complete surgical excision, highlighting the importance of complete removal and appropriate follow-up [3,11].

## Conclusions

FEPs are slow-growing benign lesions that are rarely found in the nipple. Histologically, they are characterized by a hyperplastic epidermis surrounding a fibrovascular core composed of dense collagen fibers, which may occasionally contain atypical stromal cells. This histological variability can raise concern for malignancy; therefore, biopsy and histological evaluation are essential for accurate diagnosis.

FEPs should be considered in the differential diagnosis of unusual nipple lesions, particularly when presenting as enlarging exophytic masses. Accurate diagnosis requires a multidisciplinary approach integrating clinical examination, radiological assessment, and histopathological evaluation.

Complete surgical excision remains the treatment of choice and is curative in most cases. Careful surgical planning allows preservation of nipple anatomy while achieving both diagnostic and therapeutic goals. Given the rarity of nipple FEPs, further accumulation of case reports and long-term follow-up data is required to improve understanding of their natural history, potential etiological factors, recurrence risk, and optimal management strategies.
